# Fetal demise due to stab wound with umbilical cord transection: a case report

**DOI:** 10.11604/pamj.2025.51.32.47368

**Published:** 2025-06-04

**Authors:** Skander Abid, Dhekra Toumi, Ghada Abdelmoula, Mohamed Amine Mesrati, Abdeljalil Khlifi, Haifa Bouchahda

**Affiliations:** 1 Faculty of Medicine of Sousse, University of Sousse, Sousse, 4000, Tunisia; 2 Department of Obstetrics and Gynecology, Farhat Hached University Hospital of Sousse, LR12ES03, 4000, Sousse, Tunisia; 3 Faculty of Medicine of Monastir, University of Monastir, 5019, Monastir, Tunisia; 4 Department of Gynecology and Obstetrics, Fattouma Bourguiba University Hospital, Monastir, Tunisia; 5 Department of Forensic Medicine, Tahar Sfar Hospital Mahdia, Mahdia 5100, Tunisia; 6 Department of Gynecology and Obstetrics, Tahar Sfar Hospital Mahdia, Mahdia 5100, Tunisia

**Keywords:** Fetal death, pregnancy complications, abdominal injuries, umbilical cord, case report

## Abstract

Intrauterine fetal demise due to penetrating abdominal trauma is rare, and complete umbilical cord transection is exceptionally uncommon. We report the case of a 29-week pregnant woman who sustained multiple abdominal stab wounds, leading to fetal death and complete transection of the umbilical cord. The maternal and fetal risks associated with trauma rise significantly with gestational age. Despite advances in emergency care, management of trauma in pregnancy remains complex and lacks standardized protocols. A multidisciplinary approach in specialized settings is essential to improve maternal and fetal outcomes. This case is reported due to its rarity and to underscore the importance of early recognition and coordinated intervention in traumatic injuries during pregnancy.

## Introduction

Trauma during pregnancy, although relatively uncommon, remains a leading cause of non-obstetric maternal and fetal morbidity and mortality [1]. It affects approximately 6% to 7% of all pregnancies and is associated with significant risks, particularly in the third trimester, when the uterus becomes more exposed to direct trauma [1,2]. Fetal mortality following maternal trauma ranges between 30% and 70%, depending on the severity and mechanism of injury [3]. Penetrating trauma, especially from gunshot wounds, is more frequently reported in the literature; however, cases involving stab wounds are far less common [3]. Among these, intrauterine fetal demise due to umbilical cord transection is an exceptionally rare event [4]. We report the case of a 29-week pregnant woman who sustained multiple abdominal stab wounds, resulting in intrauterine fetal death with complete transection of the umbilical cord.

## Patient and observation

**Patient information:** a 34-year-old primigravida, primiparous woman at 29 weeks of gestation, was transferred to the emergency department one hour after sustaining multiple stab wounds. She had no significant medical history, and her pregnancy had been uneventful until the incident.

**Timeline of current episode:** approximately one hour before admission, the patient, a 34-year-old primigravida at 29 weeks of gestation, was assaulted by her husband and sustained multiple abdominal stab wounds. Emergency medical services were called to the scene, and she was transferred urgently to the emergency department.

**Figure 1:**
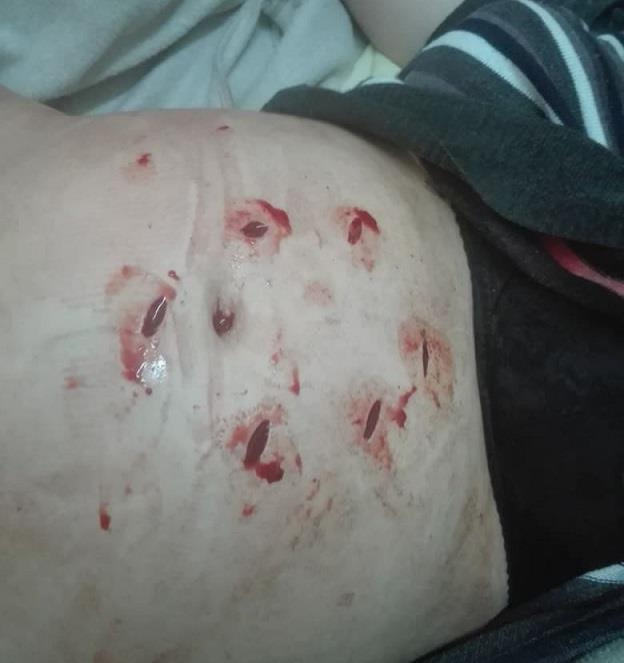
multiple abdominal stab wounds in the pregnant patient, surrounding the umbilical region

**Clinical findings:** upon admission, the patient was conscious but agitated, with pallor, cold extremities, hypotension (blood pressure: 100/60 mmHg), and tachycardia (140 bpm). Physical examination revealed two oval-shaped, red anterior cervical ecchymoses and a supraclavicular ecchymosis, consistent with manual strangulation. Abdominal examination identified six stab wounds surrounding the umbilicus, inflicted by a sharp and pointed object (Figure 1).

**Diagnostic assessment:** an emergency abdominopelvic ultrasound revealed a singleton pregnancy estimated at 29 weeks of gestation with absent fetal cardiac activity and severe oligohydramnios. A moderate hemoperitoneum was also detected. Given the patient´s hemodynamic instability, she was immediately stabilized and transferred to the operating room.

**Diagnosis:** the final diagnosis was intrauterine fetal demise due to complete transection of the umbilical cord, secondary to multiple penetrating abdominal stab wounds.

**Figure 2:**
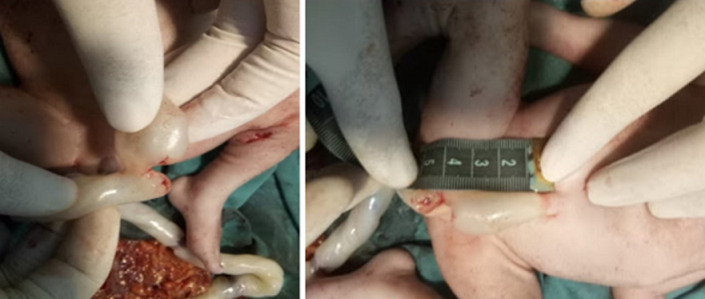
near-complete section of the umbilical cord at 4cm from its origin

**Figure 3:**
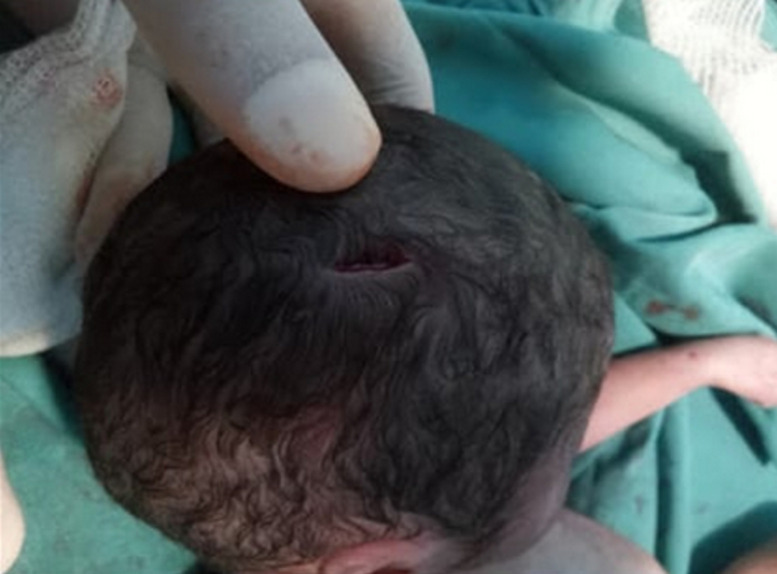
a right parietal scalp wound

**Figure 4:**
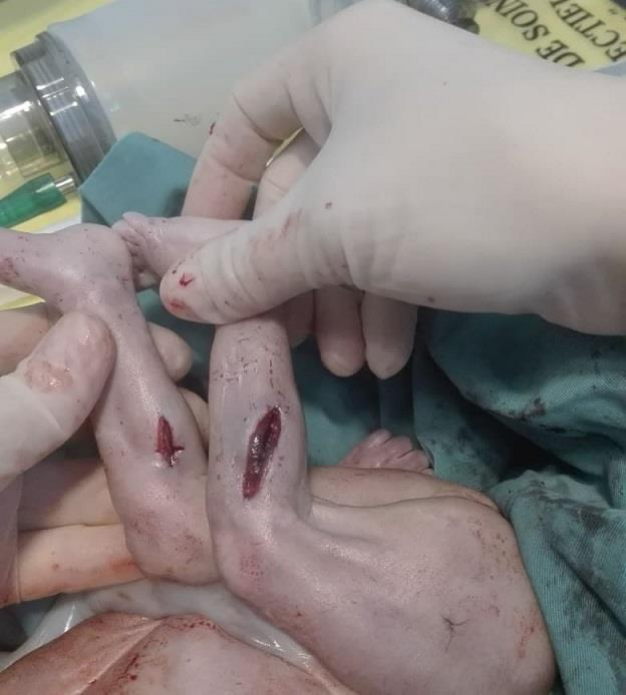
transfixing wounds on both legs

**Therapeutic interventions:** under general anesthesia, a midline laparotomy was performed. Surgical exploration revealed a moderate hemoperitoneum mixed with amniotic fluid, without evidence of bowel content leakage. The following injuries were identified: two full-thickness stab wounds to the right transverse colon, three small, clean-cut perforations of the proximal small intestine at 20cm, 30cm, and 40cm from the duodenojejunal junction, a mesenteric root hematoma and a non-expanding retroperitoneal hematoma and four bleeding uterine lacerations. A cesarean section was performed, delivering a stillborn fetus. The uterine wounds were sutured using X-shaped Vicryl 1/0 stitches. Persistent uterine atony necessitated escalating uterotonic therapy, followed by triple vascular ligation and uterine compression sutures, which failed to control the hemorrhage. Bilateral hypogastric artery ligation was ultimately required. Bowel injuries were repaired using PDS 3/0 sutures, and the retroperitoneal hematoma was left undisturbed. Intraoperative blood tests showed a hemoglobin level of 4g/dL, prompting transfusion of five units of packed red blood cells. Examination of the fetus revealed a near-complete transection of the umbilical cord 4cm from its origin (Figure 2), along with four sharp-force injuries: a 1cm, regular-edged, ecchymotic parietal scalp wound (Figure 3), a punctiform, ecchymotic mid-parietal scalp wound, a 2.2cm transfixing wound on the right leg (external) and 2cm on the internal aspect (Figure 4) and a 1.3cm transfixing wound on the left leg (external) and 1.4cm on the internal aspect (Figure 4).

**Follow-up and outcome of interventions:** postoperative recovery was uneventful, and the patient was discharged on the 10^th^ postoperative day with a scheduled follow-up in one month. The assailant (her husband) was arrested. The forensic report concluded that the cause of fetal death was massive hemorrhage due to umbilical cord transection, and the described maternal injuries were potentially fatal without urgent medical and surgical intervention. The patient had a total temporary incapacity of 60 days. At six months post-aggression, the injuries were considered healed after three months of rest, treatment, and rehabilitation. The patient reported persistent pain at scar sites and moderate post-traumatic stress disorder. The estimated permanent disability was 10%, with a moderate aesthetic impairment score (3/7) and significant physical and psychological suffering (6/7).

**Patient perspective:** six months after the traumatic event, the patient expressed deep emotional distress over the loss of her child and the violent nature of the attack. She reported persistent physical pain at the surgical scar sites and ongoing psychological distress, including anxiety and sleep disturbances. Despite medical and psychological support, she continues to struggle with the long-term consequences of the assault. However, she remains determined to rebuild her life and regain a sense of normalcy.

**Informed consent:** the patient gave informed consent.

## Discussion

Severe trauma remains one of the leading causes of non-pregnancy-related mortality in pregnant women [3]. Between 6% and 7% of pregnant women seek medical care after experiencing trauma during pregnancy, while prospective studies suggest that up to 20% may encounter traumatic events [2]. Pregnancy induces significant anatomical and physiological changes that alter the presentation and management of traumatic injuries. Early in pregnancy, the uterus remains within the pelvis, protecting the fetus from direct trauma. However, by 36 weeks of gestation, the uterus expands above the umbilicus, making it more vulnerable to injury [5]. The risk of maternal and fetal complications due to trauma increases with gestational age: 10-15% in the first trimester, 32-40% in the second, and 50-54% in the third trimester [6].

Penetrating trauma during pregnancy is rare but carries a poor prognosis for both the mother and fetus [7]. Maternal mortality following trauma can reach 10%, while fetal mortality can be as high as 70% [8]. Trauma-induced fetal mortality is often due to placental abruption, maternal hemorrhagic shock (responsible for 80% of fetal deaths), or maternal death. Although fetal trauma is uncommon, fetal death resulting from umbilical cord transection due to penetrating injury is exceedingly rare. This case highlights an unusual mechanism of fetal exsanguination, contributing valuable insight into the literature on trauma-related fetal deaths [9].

The rarity of the injury mechanism is central to this case. The umbilical cord is typically protected within the amniotic sac, making complete transection uncommon. The presence of multiple fetal stab wounds suggests a direct assault on the fetus, an occurrence rarely documented in trauma literature [10]. This underscores the importance of considering all potential mechanisms of fetal injury when evaluating pregnant trauma patients, particularly in cases involving penetrating trauma.

Management of penetrating trauma during pregnancy does not significantly differ from standard trauma protocols. However, physiological changes during pregnancy, such as increased blood volume, altered hemodynamic responses, and shifts in organ positioning, can influence injury patterns and treatment approaches [4]. Maternal stabilization remains the priority, as fetal survival is closely linked to maternal well-being. Some decisions, such as the timing of surgical interventions or the need for a perimortem cesarean section, may be challenging and require rapid multidisciplinary coordination [3].

This case also brings attention to the broader issue of intimate partner violence (IPV) during pregnancy. Trauma, particularly violence, is a leading cause of maternal mortality, and IPV is a significant contributor. Early screening and intervention programs are essential to address this public health concern. Furthermore, psychological support for pregnant women who experience violence is critical, as such trauma can lead to post-traumatic stress disorder (PTSD) and depressive disorders, affecting both maternal and fetal health [11]. A multidisciplinary approach is essential in managing pregnant trauma patients, emphasizing the need for vigilance in diagnosing rare causes of fetal death and strengthening preventive measures against domestic violence during pregnancy [11].

Healthcare providers play a crucial role in early detection through routine screening during prenatal visits, providing a safe space for victims to disclose abuse. Training medical personnel to recognize signs of violence and to offer appropriate guidance and referrals is essential. Additionally, strengthening legal frameworks to protect victims, ensuring access to emergency shelters, and providing psychosocial and legal support are critical measures.

Public awareness campaigns are also fundamental in breaking the cycle of violence, challenging societal norms that perpetuate gender-based abuse, and promoting gender equality. Community-based interventions, including education programs for both men and women, can foster cultural shifts toward nonviolence. Ultimately, eradicating violence against women requires concerted efforts from healthcare systems, policymakers, law enforcement, and civil society to create a safe and supportive environment where women can seek help without fear and receive the care and protection they need.

## Conclusion

Intrauterine fetal death due to a stab wound is an exceptionally rare event that underscores the critical issue of physical abuse during pregnancy. This case highlights the importance of considering the unique anatomical and physiological changes of pregnancy when assessing and managing trauma in pregnant women. While the management of pregnant trauma victims lacks a standardized protocol, it always necessitates a multidisciplinary approach and medical-surgical resuscitation. The key takeaway from this case is the need for heightened awareness and prompt intervention to address the potential for rare yet catastrophic injuries, including those resulting from physical abuse, during pregnancy.
